# Capillary-Regulated Carbonized Lotus Stem for Efficient Solar-Driven Water Evaporation and Purification

**DOI:** 10.3390/biomimetics11080538

**Published:** 2026-08-03

**Authors:** Zhiqiu Yuan, Xinyi Li, Shiyu Deng, Liuzhang Hu, Lijun Li, Kaijie Zhang, Pengyu Zhang, Zhi Yang, Junchao Huang, Kai Huang, Langquan Shui, Longjian Xue

**Affiliations:** 1 School of Power and Mechanical Engineering, Wuhan University, South Donghu Road 8, Wuhan 430072, China; 2023302081197@whu.edu.cn (Z.Y.); 2023302081188@whu.edu.cn (X.L.); 2023302081254@whu.edu.cn (S.D.); hlzwhu@whu.edu.cn (L.H.); lijunli@whu.edu.cn (L.L.); zkjwhu@whu.edu.cn (K.Z.); pengyuzhang@whu.edu.cn (P.Z.); 2024202080023@whu.edu.cn (Z.Y.); junchaohuang@whu.edu.cn (J.H.); 2 School of Civil Engineering, Wuhan University, South Donghu Road 8, Wuhan 430072, China; 00030505@whu.edu.cn (K.H.); l.q.shui@whu.edu.cn (L.S.)

**Keywords:** solar-driven evaporation, lotus stem, capillary regulation, biomass carbon, water purification

## Abstract

Freshwater shortages have attracted increasing global concern. Solar-driven evaporation from wastewater or seawater has emerged as a promising technology to collect fresh water; however, achieving high-evaporation efficiency remains a significant challenge. Here, we propose a capillary-regulated solar evaporator, CHALS, derived from agricultural waste—specifically, lotus stems. CHALS is constructed by the carbonization and hydrophilization of a compressed assembly of lotus stems. The compression, followed by carbonization, narrows the straight capillary channels; subsequent oxygen plasma treatment optimizes surface hydrophilicity. The two synergistic effects jointly elevate the Laplace capillary driving force to greatly accelerate internal water transportation. Meanwhile, the straight-through channels provide the shortest pathway for water transportation. Moreover, the efficient photothermal conversion raises the surface temperature of CHALS up to 65 °C. Benefiting from these synergistic effects, CHALS achieves an evaporation rate of 2.60 kg·m^−2^·h^−1^ under 1 sun irradiation. The work not only provides an agricultural waste-derived solar evaporator but also establishes a capillary-regulation strategy to boost solar evaporation efficiency.

## 1. Introduction

According to data released by the UN Food and Agriculture Organization, global per capita renewable freshwater resources have declined by approximately 7% over the past decade [1], a trend attributed to industrialization, climate change, and increasing water pollution. Expanding accessible water supplies through seawater desalination [2,3] and wastewater purification [4,5], while mitigating further contamination, represents a critical global challenge. Atmospheric water harvesting has also been considered a complementary route for decentralized freshwater production [6]. Solar-driven water purification has emerged as a promising pathway, owing to its sustainability, low operating cost, and minimal environmental footprint [7,8]. Beyond freshwater production alone, solar-driven systems have also been explored for synergistic clean water harvesting and sustainable energy production [9].

Efficient photothermal conversion and fast water transportation are the two primary technological bottlenecks for solar water evaporation [10]. The latter governs the rate at which water is absorbed and transported, while the former determines the fraction of incident sunlight transformed into heat for the evaporation of transported water. Accordingly, thermal-field regulation and system-level thermal management have become important directions for improving solar desalination and water collection performance [11,12]. Commonly employed photothermal materials include biomass, carbon-based substances, plasmonic metals, polymers, and semiconductors [13,14]. Among these, biomass, with its inherent porous architecture, has attracted significant interest due to its low cost, wide availability, and sustainability. Although untreated biomass can function as an evaporator [15], the performance can be improved by integrating darker, highly absorptive materials like tannic acid, carbon nanomaterials, metal nanoparticles, and so on [16,17,18,19,20,21]. In addition, waste-derived photothermal materials have also been explored as sustainable evaporators for solar water production [22]. For example, coating pomelo peel with ultra-black carbon yielded a water evaporation rate of 1.81 kg·m^−2^·h^−1^ under 1 sun irradiation, which is 30% higher than that of a traditional 2D evaporator [23]. However, biomass decomposes rapidly upon contact with water.

Carbonization offers an effective route with which to enhance the performance of biomass-derived evaporators. This process not only enhances environmental stability but also significantly improves broadband light absorption, boosting photothermal conversion efficiency [24,25]. Additionally, carbonization substantially reduces material density, facilitating the fabrication and deployment of lightweight devices [26]. Based on the pore geometry, biomass-derived porous carbon materials can be mainly divided into two categories. The first is a bicontinuous porous structure that is preserved from the inherent porous architecture in biomass [27,28,29,30] or formed due to the formation of carbon powder. The bicontinuous porous structure is beneficial for the adsorption of substances from the passing fluid due to the large specific surface area. On the other hand, the random and bicontinuous pores/channels significantly increase fluid resistance, reducing the efficiency of liquid transportation. The second category comprises straight-through pore structures, which are widely present in plants such as wood, reed stem, and lotus [31,32,33,34,35,36,37]. As an aquatic plant, different parts of lotus plants have been converted into carbon materials for solar evaporators. For instance, the carbonization of lotus seedpods and the connected leaf stem achieved an evaporation rate of 1.30 kg·m^−2^·h^−1^ under 1 sun illumination [31]. Taking advantage of the large surface area of the lotus leaf for light absorption and the straight channels in the stem for water transportation, researchers achieved an evaporation rate of 1.33 kg·m^−2^·h^−1^ under 1 sun irradiation [33]. The carbonization of balsa wood achieved an even higher evaporation rate of 2.13 kg·m^−2^·h^−1^ [34]. Despite these significant achievements in biomass-derived carbon materials, further improvements in evaporation efficiency are still needed and remain a considerable challenge.

Here, we propose a capillary-regulation strategy to enhance solar-driven evaporation using carbonized lotus stems. The lotus stems are assembled in parallel and compressed at a certain pressure, followed by carbonization and hydrophilization. For convenience, the resulting material is referred to as CHALS. The reduced channel diameter, achieved by compression, and the surface modification greatly increase the Laplace pressure in the channels, enhancing water transport, and increasing the evaporation rate to 2.60 kg·m^−2^·h^−1^ under 1 sun illumination. Moreover, CHALS is demonstrated to be a reliable device with which to collect clean water from wastewater. Therefore, the design strategy establishes a promising solution with which to boost the solar evaporation of biomass derivatives.

## 2. Materials and Methods

### 2.1. Materials

Dried lotus stems were collected from local farmers in Hubei Province, China. Cyanoacrylate adhesive was obtained from local supermarkets. Methyl orange, crystal violet, and malachite green were purchased from Shanghai Lingfeng Chemical Reagent Co., Ltd., Shanghai, China. All chemicals were used as received, without further purification.

### 2.2. Preparation of CHALS

Dried lotus stems were cut into segments of approximately 3 cm in length and assembled into blocks using a home-made rectangular mold that consisted of a grooved base (3 cm × 8 mm) and a plunger (height: 1.1 cm). For each layer, eight stem segments were aligned along the mold and uniaxially compressed using a universal testing machine (5942, Instron, Norwood, MA, USA) under pressures ranging from 10 to 50 MPa for 5 min. Cyanoacrylate adhesive was applied on top of each layer before the following layer of assembled stem segments. This layer-by-layer procedure was repeated until dense lotus-stem blocks with approximate dimensions of 3 cm × 3 cm × 5 cm were obtained.

The assembled blocks were subsequently carbonized in a tubular furnace (OTF-1200X, KJ, Hefei, China) under a continuous argon flow at temperatures ranging from 600 to 800 °C for 4 to 5 h. Finally, the carbonized blocks were treated with oxygen plasma in a plasma cleaner (Flecto 10, Plasma Technology, Herrenberg, Germany) at a power of 100 W and a pressure of 0.8 Pa for 3 min.

### 2.3. Characterization

The microstructures of samples were examined using field-emission scanning electron microscopy (JSM-IT300, JEOL, Tokyo, Japan) after sputter-coating with a thin gold layer. Surface wettability was evaluated using a contact-angle goniometer (OCA25, DataPhysics, Filderstadt, Germany). Thermal behavior under simulated solar irradiation was recorded with an infrared thermal imager (TiX501, Fluke, Everett, WA, USA) using a xenon solar simulator (SS-100A, Beijing Sanyou Dingsheng Technology Co., Ltd., Beijing, China). Surface functional groups were characterized by in situ diffuse reflectance infrared Fourier transform spectroscopy (Nicolet iS50, Thermo Fisher Scientific, Waltham, MA, USA) between the range of 4000 to 400 cm^−1^ and confocal Raman microscopy (XploRA Plus, HORIBA Jobin Yvon, Palaiseau, France). Water before and after evaporation was analyzed using a UV–Vis spectrophotometer (UV-2550, Shimadzu, Petaling Jaya, Malaysia) between a wavelength range of 200 to 800 nm.

Each CHALS sample was wrapped with a 2 cm thick layer of polystyrene foam to provide buoyancy and thermal isolation from the bulk water. A xenon lamp was employed to simulate solar irradiation at an intensity of 1 sun with the light beam vertically incident on the sample surface. Evaporation performance was evaluated for 1 h on three independently fabricated parallel samples, with the measurement repeated three times for each sample. The evaporation rate was calculated from the mass change of water before and after evaporation, normalized by the effective evaporation area and testing time. The capillary water-transport performance of carbon powder, uncompressed carbonized lotus-stem segments; CHALS was evaluated under identical conditions. The initial time was defined as the moment when the bottom of the sample first contacted water; the endpoint was defined as the moment when water first reached the upper surface. The measured time for transportation was normalized by the corresponding vertical distance.

Data analysis, statistical analysis (One-way ANOVA), and graph plotting were performed using Origin (Version 2024, OriginLab, Northampton, MA, USA). The pore sizes from SEM images were measured using ImageJ software (Version 1.53m, National Institutes of Health, Bethesda, MD, USA).

## 3. Results and Discussion

### 3.1. Construction of CHALS

**Figure 1:**
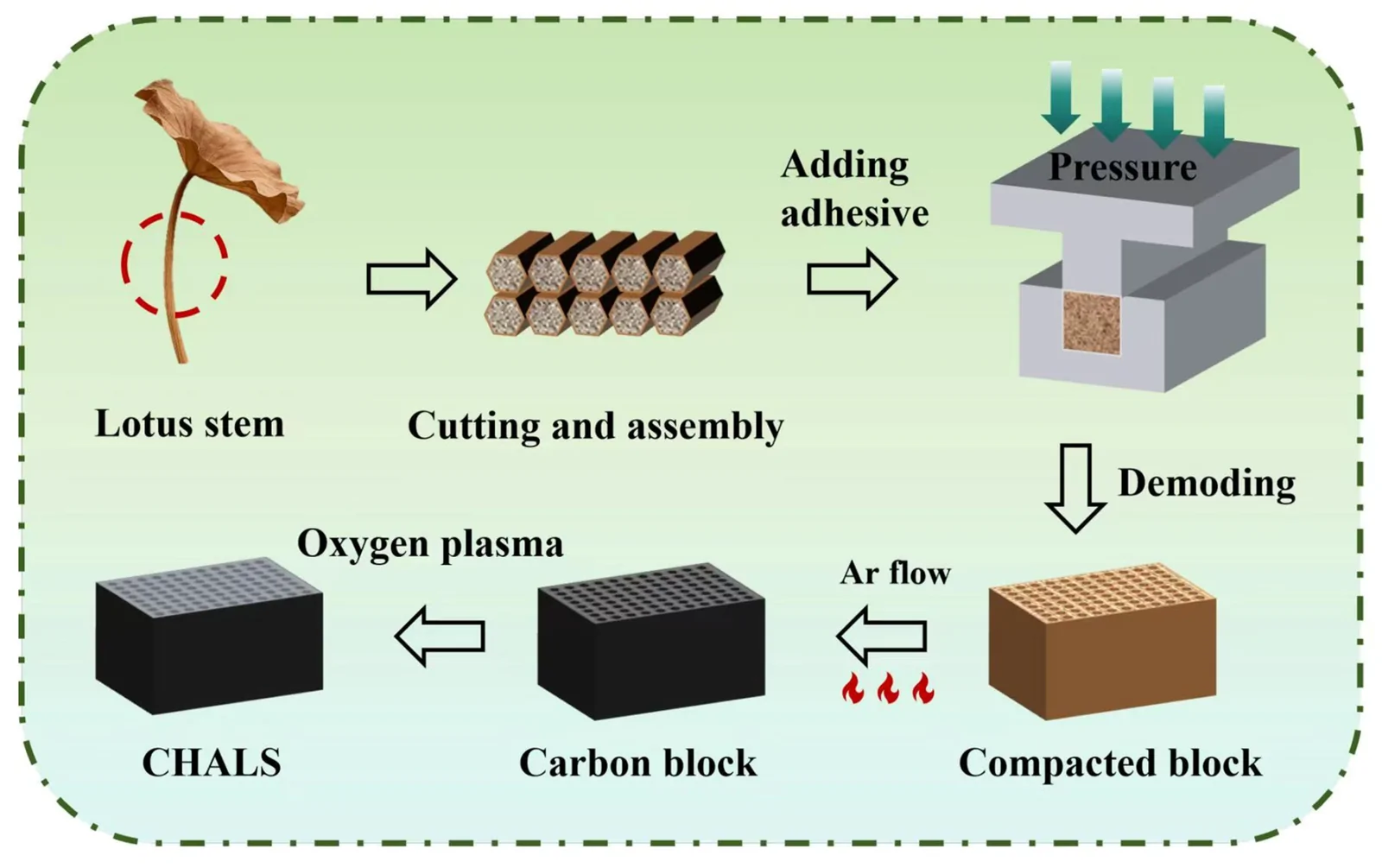
Fabrication process of CHALS. The red dashed circle indicates the harvested section of lotus stem and teal arrows represent the applied compression pressure.

**Figure 2:**
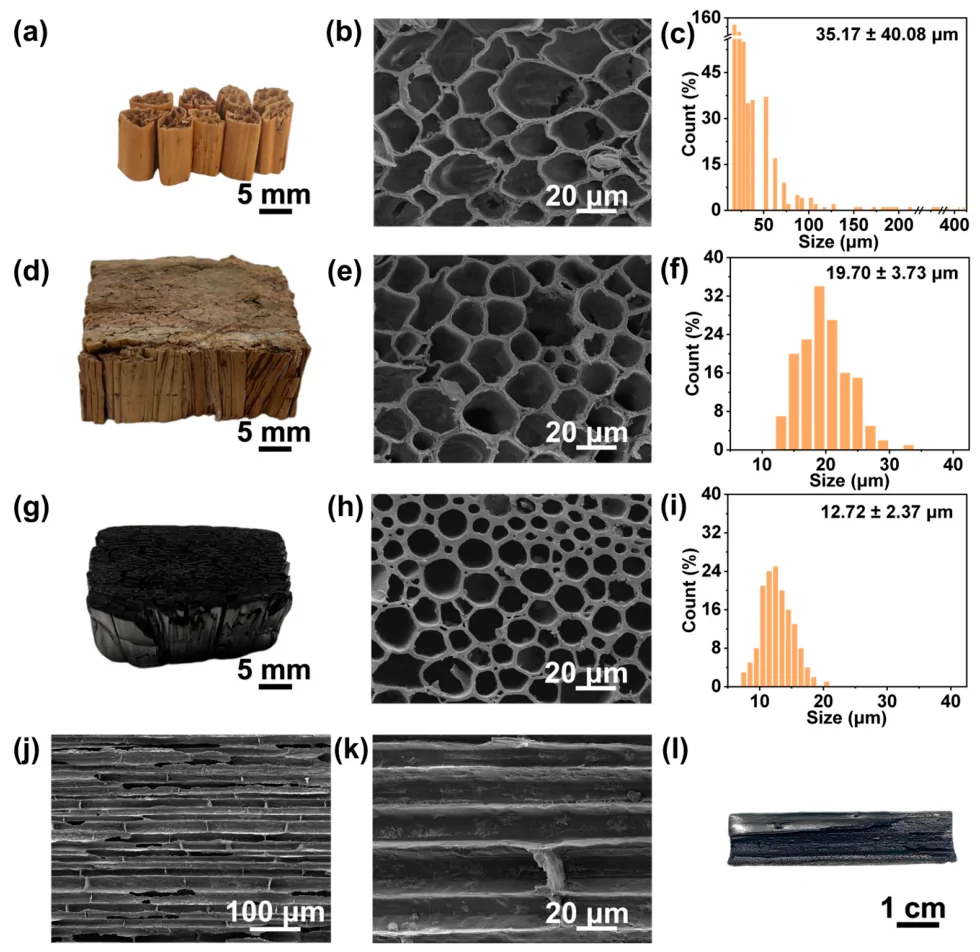
(**a**,**d**,**g**) optical images; (**b**,**e**,**h**) SEM images; and (**c**,**f**,**i**) corresponding pore size distributions of (**a**–**c**) original lotus stems; (**d**–**f**) the block of lotus stems compressed at 30 MPa; and (**g**–**i**) CHALS prepared under 30 MPa compression and carbonized at 700 °C, respectively. (**j**,**k**) SEM images of the longitudinal-section of CHALS; and (**l**) optical image of uncompressed carbonized lotus stem.

Lotus is a very important agricultural plant in Hubei Province, China and lotus stems and leaves are major agricultural wastes. Here, CHALS was prepared from the dried lotus stems. The preparation process was quite straightforward (Figure 1, please refer to details in the experimental section). The original dried stems are highly porous (Figure 2a,b), showing polygonal pores with a mean diameter of 35.17 ± 40.08 μm (Figure 2c). The compression, such as under a pressure of 30 MPa, greatly reduced the pore diameter to 19.70 ± 3.73 μm, while preserving the pores (Figure 2d–f). The compression not only pre-defined the size of the channels but also established a constrained framework for subsequent carbonization. The carbonization of the compacted block at 700 °C, in a protective atmosphere, resulted in a carbon block. The carbon block was treated with oxygen plasma, yielding CHALS. Notably, the pores in CHALS have a near-round shape (Figure 2g,h) with a sharply reduced pore diameter of 12.72 ± 2.37 μm (Figure 2i), which is 63.83% of that found in the uncarbonized and uncompressed stems. Moreover, there are small pores, with diameters as small as 7.03 μm, formed in the joint areas among big channels. Meanwhile, the channel walls are rather smooth, with the presence of some porous, bamboo septum-like structures within the channels (Figure 2j,k). To provide an appropriate reference for evaluating the effect of compression on the channel structure after carbonization, an uncompressed lotus-stem sample was carbonized under the same conditions (Figure 2l). The macroscopic central hollow cavities, in which no observable capillary rise occurred under the present experimental conditions, were excluded from the channel-size analysis. The remaining water-accessible channels exhibited an overall mean diameter of 17.40 ± 17.85 μm. The relatively large standard deviation reflects the broad and heterogeneous channel-size distribution of the uncompressed, carbonized lotus stem.

**Figure 3:**
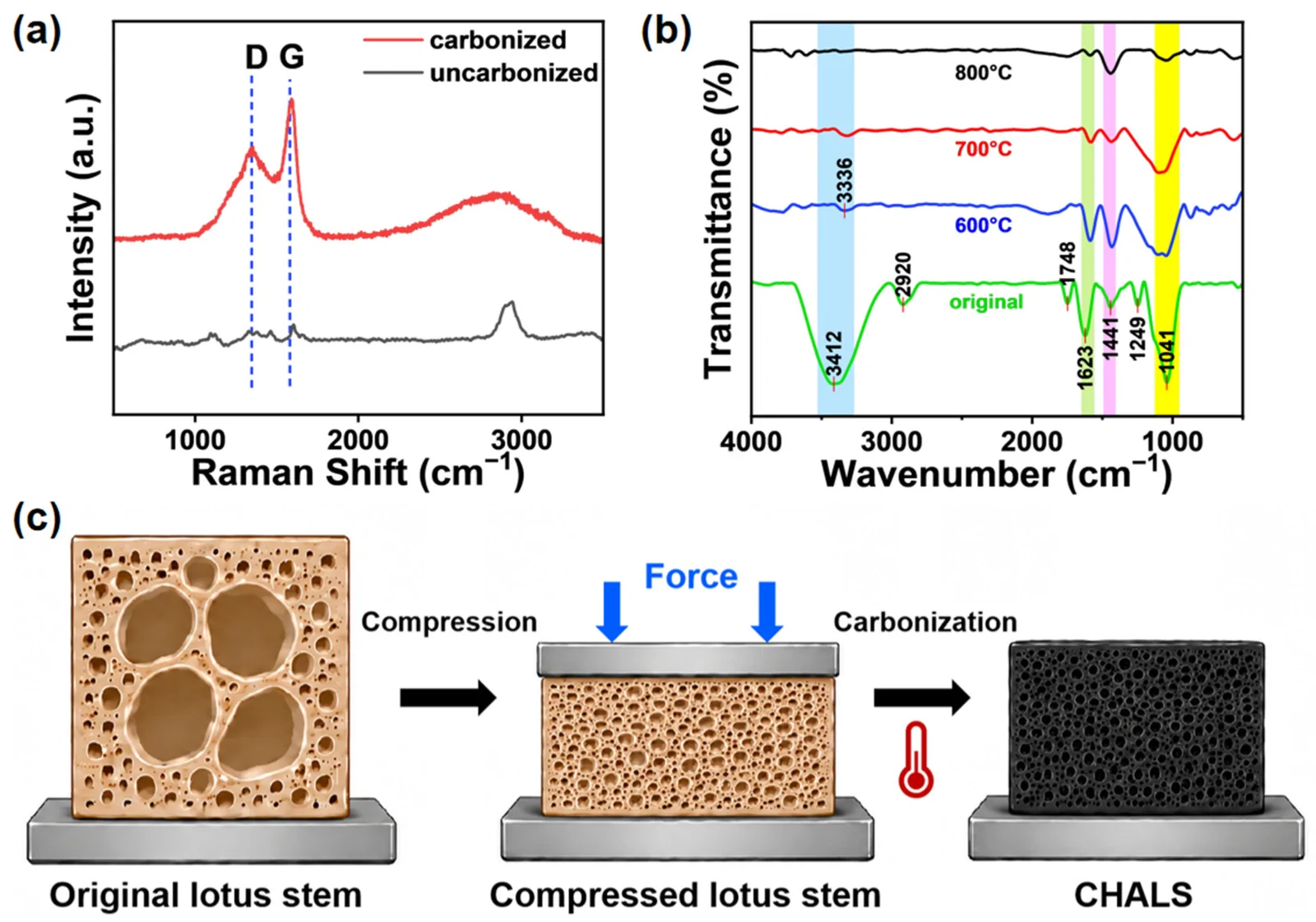
(**a**) Raman spectra of original lotus stem and that carbonized at 700 °C; (**b**) FTIR spectra of original lotus stems and that carbonized at 600, 700, and 800 °C; and (**c**) schematic illustration of the evolution of the lotus-stem pore structure during compression and subsequent carbonization.

The chemical component of CHALS was systematically characterized. The Raman spectrum (Figure 3a) of the untreated lotus stem showed multiple characteristic peaks in the regions of 2950–2850 cm^−1^ (saturate C–H stretching vibrations), 1740–1720 cm^−1^ (C=O stretching vibration of aldehyde), and 1690–1630 cm^−1^ (C=O stretching of amide). Additionally, bands in the ranges of 1470–1350 cm^−1^ and 1350–1000 cm^−1^, attributed to aliphatic C–H bending vibrations and various C–O stretching vibrations, respectively, were observed, indicating the presence of abundant aliphatic and oxygen-containing organic components. After carbonization, these organic signals were greatly reduced. The intensity ratio of the D and G bands was 0.64, suggesting the formation of a carbon structure with a moderate degree of graphitization and a low-to-moderate defect density [38].

The proper carbonization was further demonstrated by FTIR spectroscopy (Figure 3b). The typical absorptions of biomass, such as the vibrations of the methylene group at 2920 cm^−1^ and carbonyl groups at 1748 cm^−1^, completely vanished when the carbonization temperature reached 600 °C. Meanwhile, the band at 1249 cm^−1^, which is assigned to C–O stretching vibrations associated with aromatic ether (Ar–O–C), also disappeared. In contrast, the stretching vibration of the hydroxyl group at 3412 cm^−1^, vibrations of the methylene group at 1441 cm^−1^, C–O stretching vibrations associated with aliphatic ether groups at 1041 cm^−1^, and the absorption band at 1623 cm^−1^, which is attributed to the aromatic ring skeletal stretching vibration, were gradually reduced but remained under temperatures up to 800 °C. These features suggest that the oxygen-containing functional groups and aliphatic hydrocarbon structures in the lotus stems underwent progressive thermal decomposition and elimination, accompanied by the concurrent development of a more ordered graphite-like microcrystalline structure [39,40,41].

Mechanical compression and carbonization contribute synergistically to the shape refinement and size reduction of the pores in CHALS. Mechanical compression physically pre-defined the geometry/size of the channels. During carbonization, driven by the minimization of surface energy, the rearrangement of carbon atoms into an aromatic skeleton resulted in channels with a rounder profile (Figure 3c) [42]. Meanwhile, pore-wall smoothing and curvature homogenization occurred synchronously (Figure 2g,h). Moreover, the mild carbonization at 600–800 °C and the slow heating rate are conducive to preserving the porous framework [43]. Therefore, “compression pre-forming” and “carbonization-driven structural refinement” cooperatively govern the overall structural evolution of CHALS, enhancing the regularity of the channels.

### 3.2. Evaporation Performance and Mechanisms

**Figure 4:**
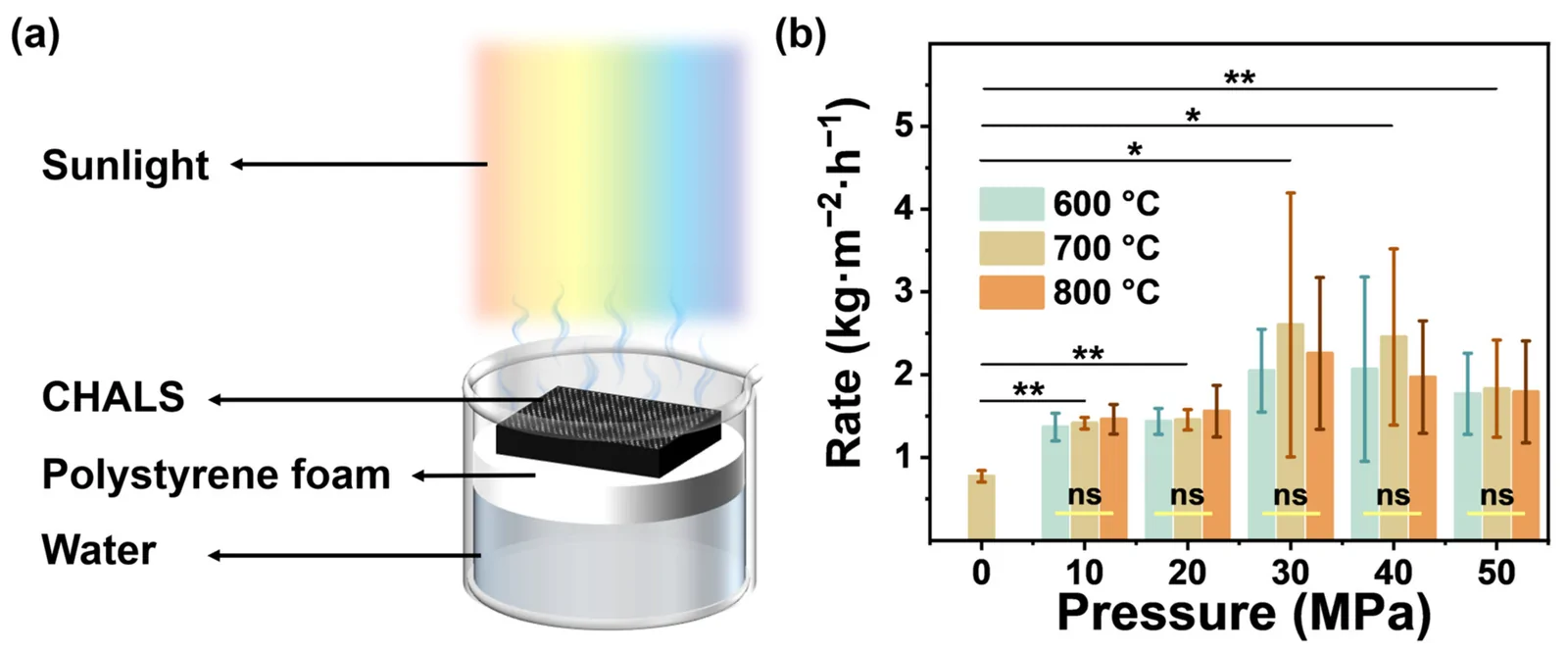
(**a**) Schematic illustration of the solar-driven interfacial evaporation device with CHALS as the key element (the colored bands represent the simulated broadband sunlight); and (**b**) influences of compression pressure on the solar evaporation rate in CHALS prepared under different carbonization temperatures. Data in (**b**) are the mean values of at least three measurements. All data were corrected by subtracting the natural evaporation in the dark. The error bars indicate standard deviations. One-way ANOVA results are presented, with significance indicated as * *p* < 0.05, ** *p* < 0.001 (ns for not significant (*p* > 0.05)).

The influences of compression pressure and carbonization temperature on the evaporation performance of CHALS were examined using a homemade interfacial evaporation system under 1 sun irradiation (1 kW·m^−2^) (Figure 4a). Uncompressed lotus stems, carbonized at 700 °C, were used as the reference, showing an evaporation rate of 0.77 kg·m^−2^·h^−1^. The compression of 10 MPa significantly increased the evaporation rate to 1.44 kg·m^−2^·h^−1^. A further increase in the compression pressure, to 30 MPa, sharply elevated the evaporation rate to 2.60 kg·m^−2^·h^−1^ (Figure 4b). A further increase in pressure up to 50 MPa, however, slightly reduced the evaporation rate. The highest evaporation rate was found at a carbonization temperature of 700 °C and a compression pressure of 30 MPa. Through significance testing (one-way ANOVA), a marked difference was observed between the uncompressed group and the compressed groups (*p* < 0.05), indicating that the compression step exerts a considerable influence on the overall evaporation rate, while no significant difference was observed in the experimental data for CHALS samples carbonized at different temperatures within the same pressure group.

**Figure 5:**
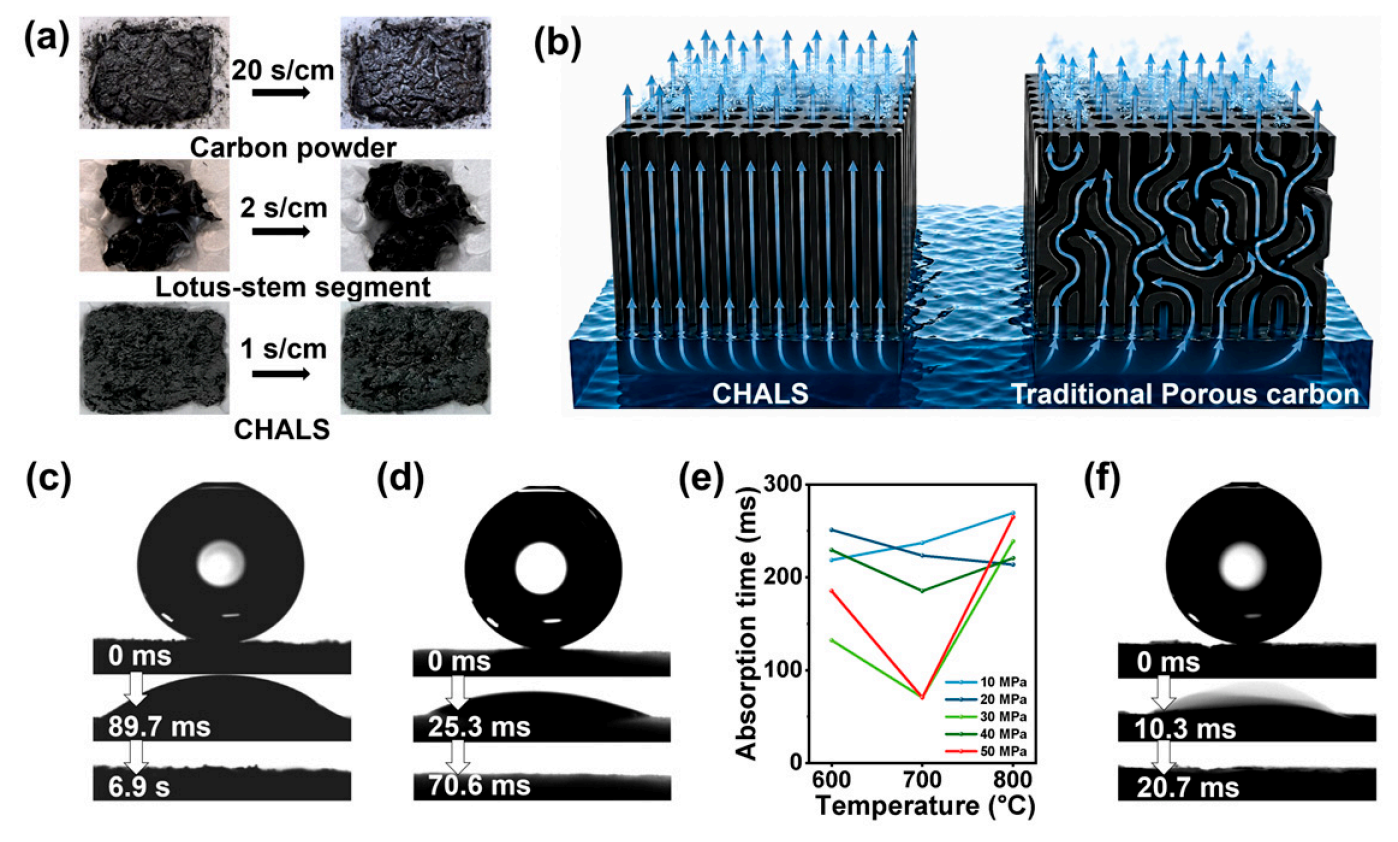
(**a**) Comparison of water transport in carbon powder, uncompressed carbonized lotus-stem segments, and CHALS. For each material, the left image shows the sample before contact with water, whereas the right image shows the sample after contact with water, when water had been transported to the upper surface; (**b**) schematic drawing of straight channels in CHALS in comparison with random pores in traditional biomass-derived carbon materials. The arrows indicate the pathway for water transportation; (**c**,**d**) evolution of a water droplet on (**c**) compressed lotus-stem block (30 MPa) and (**d**) compressed lotus-stem block (30 MPa) carbonized at 700 °C; (**e**) influence of carbonization temperature on the time for the completed absorption of a water droplet on carbonized block prepared under different compression pressures; and (**f**) evolution of a water droplet on CHALS prepared at a compression pressure of 30 MPa and a carbonization temperature of 700 °C.

The optimal performance of CHALS results from a synergistic interplay of microstructure, surface chemistry, and photothermal properties. The normalized water-transport time decreased from 20 s·cm^−1^ for carbon powder to 2 s·cm^−1^ for the uncompressed carbonized lotus-stem segment and further to 1 s·cm^−1^ for CHALS, demonstrating the superior water-transport capability of CHALS (Figure 5a). Macroscopically, the bulk water-transport speed of CHALS is twice as fast as that of uncompressed carbonized lotus-stem segments; microscopically, the droplet absorption rate is up to 3.4 times higher, which strongly demonstrates the critical promotion effect of oxygen plasma hydrophilization on water delivery. Powder-based biomass-derived carbon materials generally form disordered porous structures and tortuous interparticle pathways. Such disordered porous structures are beneficial for the absorption of pollutants from fluids because they have long transport pathways and a large surface area; however, these are unfavorable for fluid-transport efficiency. In contrast, the channels in CHALS pass directly from one side to the other, preserving the pore structure of the lotus stems and providing relatively direct and low-tortuosity pathways for water transport (Figure 5b).

Considering the channels as capillaries, Laplace pressure (*P*) drives water to pass through:

$$P=\frac{2\gamma \cos\theta }{r}$$

where *γ*, *θ*, and *r* are the surface tension of water, contact angle of water on the carbon, and mean radius of the channels in CHALS, respectively. To evaluate the effect of compression on capillary pressure after carbonization, the uncompressed lotus-stem sample, carbonized at 700 °C, was selected as the reference sample. The uncompressed carbonized lotus stem contained macroscopic, central hollow cavities surrounded by water-accessible channels with a broad size distribution. The macroscopic, millimeter-scale central hollow cavities were excluded from the channel size statistics, as no observable capillary rise could be generated within these large cavities under our experimental conditions. Only micron-scale water-accessible capillary channels were counted for the radius calculation. Water-uptake observations indicated that no observable capillary rise occurred in the macroscopic central hollow cavities under the present experimental conditions. These cavities were, therefore, excluded from the channel-size analysis. Statistical analysis of the remaining water-accessible channels yielded an arithmetic mean channel diameter of 17.40 ± 17.85 μm and a corresponding arithmetic mean channel radius of 8.70 ± 8.93 μm. The relatively large standard deviation reflects the broad and heterogeneous channel-size distribution of the uncompressed carbonized sample. During compression, the macroscopic central hollow cavities largely collapsed, while the surrounding water-accessible channels were retained and narrowed. In comparison, CHALS prepared by compression at 30 MPa and carbonization at 700 °C exhibited a mean channel diameter of 12.72 ± 2.37 μm and a corresponding mean channel radius of 6.36 ± 1.19 μm.

Relative to the uncompressed carbonized reference sample, this represents a reduction of approximately 26.90% in the arithmetic mean channel radius. If only the change in channel radius is considered, the corresponding theoretical capillary-pressure enhancement factor is 1.36. Because both samples possess highly porous and rapidly absorbing surfaces, water droplets were absorbed into the channels almost immediately upon contact. Therefore, channel narrowing and plasma-enhanced wettability jointly promote capillary water uptake in CHALS. Because reliable numerical contact angles could not be obtained, only the contribution of the channel-radius reduction is quantified, whereas the wettability contribution is discussed qualitatively.

Upon carbonization at 700 °C (compressed at 30 MPa), a water droplet was completely absorbed into the carbon block within 70.6 ms, which is significantly shorter than the 6.9 s needed for the compressed stem block (Figure 5c,d). Carbonization induced structural shrinkage of the pores and altered the surface chemistry of the material. Therefore, the accelerated droplet absorption observed after carbonization is consistent with the combined effects of the reduced pore radius and changes in surface wetting behavior, rather than being attributed exclusively to pore-size reduction. Increasing the compression pressure or carbonization temperature, however, did not result in a monotonic increase in the absorption speed. The fastest absorption was found in the carbon block compressed at 30 MPa and carbonized at 700 °C (Figure 5e). This nonmonotonic behavior may arise from the competing effects of the driving force, surface area of pores, and surface hydrophilicity.

Hydrophilicity of the surface is beneficial for water uptake. Oxygen-plasma treatment generates carboxyl, hydroxyl and carbonyl groups on the surface of CHALS, which greatly reduces the water contact angle (*θ*) on the surface. The reduced θ also contributes to an increase in Laplace pressure, facilitating water uptake and transportation. As a result, a water droplet was completely absorbed into CHALS within 20.7 ms (Figure 5f), exhibiting an absorption time more than 330 times faster than that of the lotus-stem block (uncarbonized and not treated with oxygen plasma). These results suggest that carbonization effectively enhanced the water absorption kinetics and that hydrophilic treatment could further improve the water transportation.

**Figure 6:**
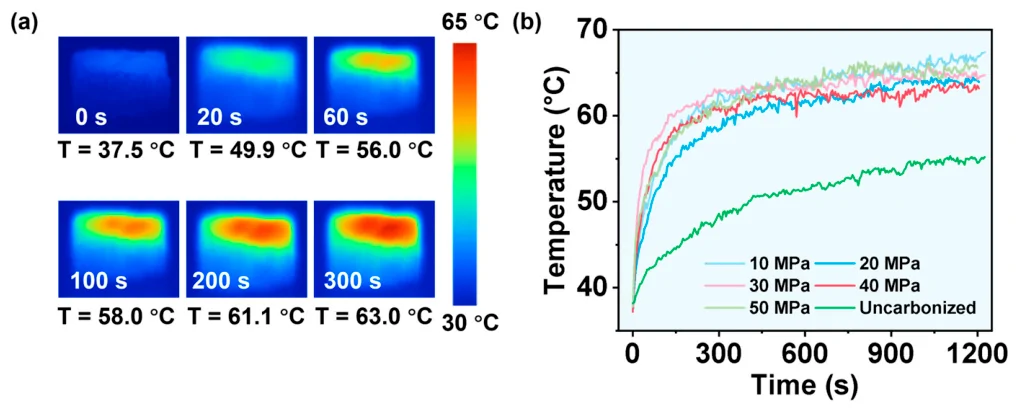
(**a**) Representative infrared images of CHALS (30 MPa compression and 700 °C carbonization) under 1 sun irradiation; and (**b**) evolution of surface temperatures of CHALS samples carbonized at 700 °C and under different compression pressures under 1 sun irradiation.

Carbonization formed an aromatic carbon framework with strong broadband light absorption, which is crucial for solar evaporation. The photothermal performance was evaluated by monitoring the surface temperature under simulated solar irradiation. The surface temperature of CHALS (carbonized at 700 °C and compressed at 30 MPa) rose rapidly upon illumination, reaching an equilibrium temperature of about 63.0 °C within ~300 s (Figure 6a). The equilibrium temperatures for CHALS samples prepared at different compression pressures ranged between 60 and 65 °C, showing minor differences (Figure 6b). In contrast, the uncarbonized lotus-stem block exhibited a significantly lower equilibrium temperature of only 49.9 °C under identical conditions, suggesting that carbonization is the primary factor for the effective photothermal response. Furthermore, infrared imaging confirmed that the surface temperatures of all carbonized samples were uniform and significantly higher than the ambient temperature, further verifying the efficient photothermal conversion.

### 3.3. Potential for Practical Application

**Figure 7:**
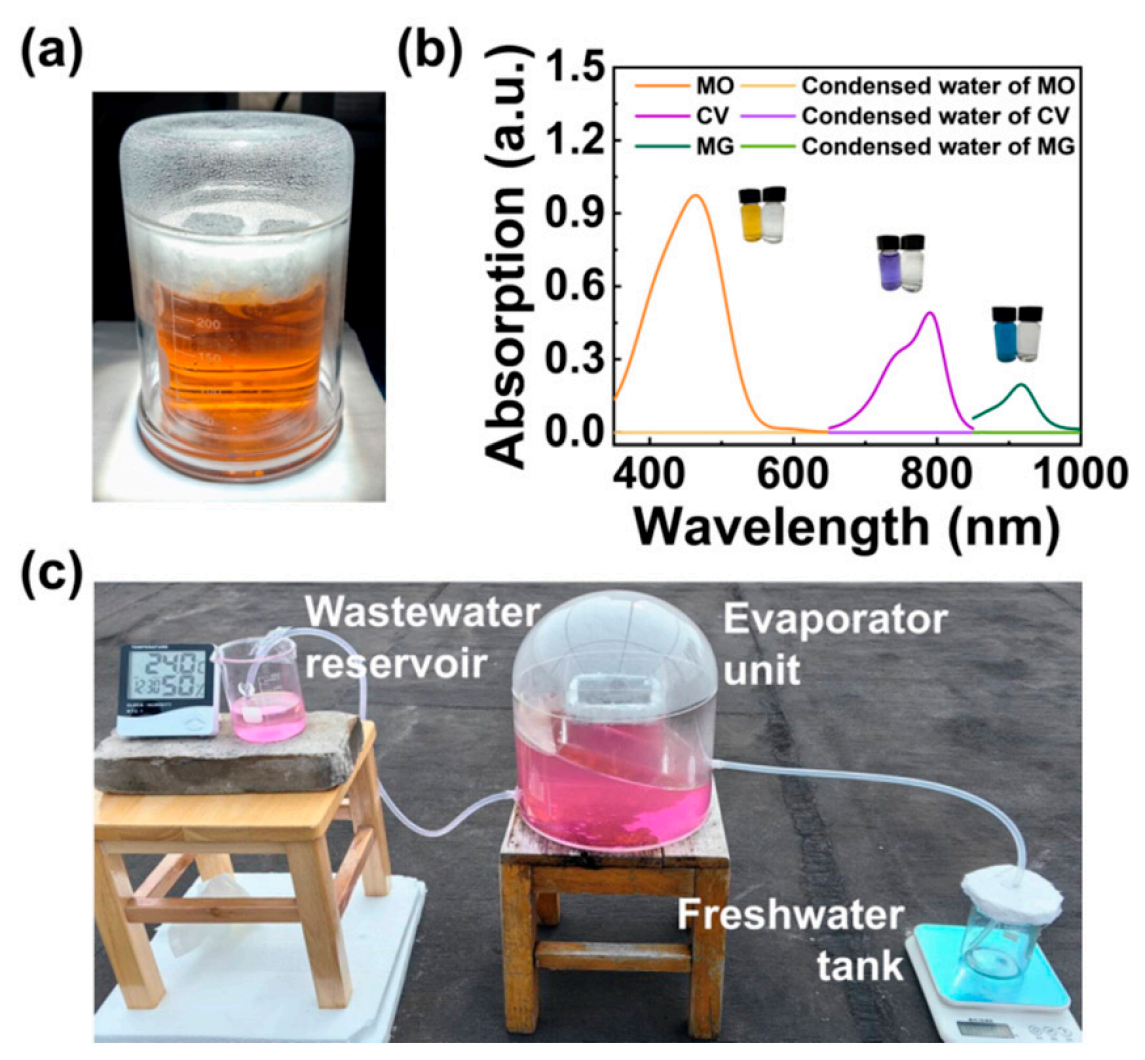
(**a**) Home-made lab-scale solar water purification setup; (**b**) UV–Vis absorption spectra and corresponding photographs of methyl orange (MO, orange curve), crystal violet (CV, purple curve), and malachite green (MG, green curve) solutions before and after treatment of solar evaporation; and (**c**) photograph of the outdoor solar evaporator prototype.

The potential practical applications of CHALS were demonstrated. A laboratory-scale solar water purification setup, consisting of a solar interfacial evaporation system, a condensation cover, and a water collection trough, was constructed (Figure 7a). Under simulated 1 sun illumination, water evaporation generated by CHALS condensed onto the condensation cover and was subsequently collected as clean condensate. Methyl orange (MO), crystal violet (CV), and malachite green (MG) solutions (50 mg·L^−1^) were used to simulate wastewaters, assessing the evaporator’s purification capability for organic contaminants. While the original dye solutions exhibited characteristic absorption peaks (MO: ~464 nm; CV: ~590 nm; MG: ~617 nm), the collected water was clear and colorless with no absorption at the corresponding wavelengths (Figure 7b). This clearly indicates that CHALS can effectively produce highly purified water through solar-driven evaporation. The residual absorbance of the condensed water was nearly indistinguishable from the instrumental baseline noise (9 × 10^−4^ Abs), indicating that the dye concentrations were essentially below the detection limit. This exceptional purification performance is fundamentally attributed to the non-volatile nature of these organic dye salts, which cannot co-vaporize with water molecules at the operating temperature (~65 °C), leaving them entirely trapped within the carbon matrix.

To demonstrate the feasibility of this technology in a real-world environment and visually demonstrate its operation, a pink acrylic pigment solution was used to simulate colored wastewater in a prototype system (Figure 7c). The system consisted of a wastewater reservoir, the evaporator unit with featured enlarged evaporation and condensation areas, and a freshwater tank. By maintaining a balanced water level between the wastewater reservoir and the evaporator unit, self-replenishment of the wastewater was achieved. An inclined plate integrated inside the evaporator unit guided the condensed water directly into the freshwater tank. The continuous outdoor evaluation was conducted from 9:00 to 17:00 on a cloudy and windy day. At midday (12:30), the ambient temperature and relative humidity were recorded as 24 °C and 50%, respectively. Although the real-time solar irradiance was not explicitly measured due to equipment limitations, the system demonstrated reliable operation tracking the natural solar cycle. Notably, the maximum evaporation rate was achieved between 12:00 and 13:00, reaching 0.87 kg·m^−2^·h^−1^, successfully verifying its practical capability for clean water collection in open areas.

## 4. Conclusions

A solar evaporator, CHALS, possessing an evaporation rate of 2.60 kg·m^−2^·h^−1^ under 1 sun irradiation, was successfully constructed from an agricultural waste—lotus stem. The lotus stems were assembled along one direction and compressed into a block, followed by carbonization in an inert gas flow. The straight channels inside the lotus stems were preserved, while the diameter of these channels was greatly reduced due to compression and carbonization. The treatment with oxygen plasma converted the surface of the carbon block into a hydrophilic surface, yielding CHALS. The reduced channel size and the hydrophilic surface greatly increased the Laplace pressure, facilitating the water uptake. Meanwhile, the straight channels through CHALS provided relatively short distances for water transport. In addition, the surface temperature of CHALS can easily exceed 60 °C. The large driving force, the short water-transport distance and the high surface temperature are thus responsible for the high solar evaporation rate. The function of CHALS was further demonstrated with simulated wastewater under outdoor conditions. The work here not only provides an effective solar evaporator made of agricultural waste but also proposes a capillary-regulation strategy with which to enhance solar evaporation.

## Data Availability

Data are contained within the article.
